# Soil organic carbon dynamics matching ecological equilibrium theory

**DOI:** 10.1002/ece3.4586

**Published:** 2018-10-18

**Authors:** Tancredi Caruso, Franciska T. De Vries, Richard D. Bardgett, Johannes Lehmann

**Affiliations:** ^1^ School of Biological Sciences Institute for Global Food Security Queen's University of Belfast Belfast UK; ^2^ School of Earth and Environmental Sciences Michael Smith Building The University of Manchester Manchester UK; ^3^ School of Integrative Plant Science Cornell University Ithaca New York

**Keywords:** dynamics, equilibrium, soil continuum model, soil organic C

## Abstract

The persistence of soil organic carbon (SOC) has traditionally been explained as a combination of recalcitrance properties and stabilization processes, which lead to the formation of complex organic compounds. However, recent conceptual advances and experimental evidence challenge this view. Here, we test these conceptual advances using a dynamic equilibrium theory of SOC founded on classic ecological theory. We postulate that the persistence of SOC is an equilibrium point where SOC losses resulting from continuous decomposition and SOC gains due to SOC protection are balanced. We show that we can describe the temporal dynamics of SOC remarkably well (average and median *R* ^2^ = 0.75) in publicly available SOC time series from experiments that investigated the effects of agricultural practices in arable soils. The predictive power of our simplistic model is not meant to compete with that of current multi‐pool SOC models or recent developments that include microbial loops. The simplicity of our analysis can, however, show how the conceptual distinction between the forces that control SOC loss and gain, and their equilibrium, can shed light on SOC dynamics. Specifically, our analysis shows that, regardless of specific mechanisms, the persistence of SOC will depend on the ultimate equilibrium between SOC gains and losses, which may depend on environmental (e.g. temperature) and ecological (e.g. spatially structured microbial activities) factors and the relative roles of these factors. Future experimental studies should quantify these roles to formulate a new generation of SOC dynamics model.

## 1 INTRODUCTION

Soil plays an essential role in the global carbon (C) cycle acting as both a source and sink of organic C (Bender, Wagg, & van der Heijden, 2016; Lal, 2004, 2008; Osuri et al., 2016; Schuur et al., 2015). Soil contains three times more organic C than is contained in plants and the atmosphere, and while soil is classically viewed as a C sink on a global scale there is concern that climate and land use change will turn it into a C source (Bardgett, Freeman, & Ostle, 2008; Bond‐Lamberty, Bailey, Chen, Gough, & Vargas, 2018; Cotrufo, Wallenstein, Boot, Denef, & Paul, 2013; Lal, 2004). Such a source‐sink switch could not only be brought about by a change in the physical and chemical state of soil, but also through changes in soil biota and their interactions with plants (Bender et al., 2016; Davidson & Janssens, 2006; Osuri et al., 2016). Indeed, soil biota plays a pivotal role in soil C dynamics, especially in relation to stabilization of soil organic matter (SOM) and persistence of soil organic C (SOC).

The traditional view is that dead plant material and other biological materials are degraded by soil organisms into organic compounds, such as large humic substances that are highly resistant to further decomposition, thereby stabilizing SOM (Brady & Weil, 1996; Tan, 2014). Also, the chemical properties of organic inputs to soil have traditionally been considered to be the starting point for estimating C turnover rate because they determine the resistance of soil organic compounds to degradation (a property loosely definable as “recalcitrance”). Accordingly, current models of C dynamics (there are at least 30, as reviewed in Falloon & Smith, 2009) explicitly incorporate the traditional ideas of recalcitrance (i.e. resistance to degradation due to chemical properties) and chemical stabilization (due to abiotic and biotic formation of humic substances). For example, two of the most widely used models, RothC (Coleman & Jenkinson, 1996) and CENTURY (e.g. Parton, 2010; Parton, Schimel, Cole, & Ojima, 1987; Parton et al., 1994) are both founded on the traditional concepts of SOM recalcitrance and SOM stabilization via humification. RothC focuses primarily on soil processes to predict SOM and SOC temporal trajectories (i.e. it does not model aboveground ecosystem processes), whereas CENTURY includes both soil processes and aboveground primary production in the model. Both models account for varying quality of SOC inputs. For example, RothC uses the ratio of decomposable to resistant plant material as a driver of SOM decomposition rates, while CENTURY uses the lignin to N ratio, which determines in which proportion litter contributes to more resistant and more labile SOM compartments. RothC and CENTURY both use clay content as major variable to partition fluxes of C between various discrete SOM pools, with each pool characterized by a specific decomposition rate and the lowest decomposition rates are assigned to the humified organic matter.

Traditional models of SOM neglect to explicitly include that decomposition of SOM is dependent on ecological context, especially microbial distribution and activity, and the presence of substrates that activate decomposition processes. For example, even compounds such as lignin, traditionally considered very recalcitrant, can have a turnover rate higher than that of bulk SOM (Schmidt et al., 2011). Conversely, compounds that are traditionally considered labile, such as root exudates and microbially derived cytoplasmic materials, can persist for decades and are increasingly recognized as a key constituent of relatively persistent SOM (Kallenbach, Frey, & Grandy, 2016). Also, the formation of aggregates, together with the adsorption of simple and small compounds into mineral surfaces, plays a fundamental, yet underestimated, role in SOM protection (Lehmann & Kleber, 2015). These findings, along with recent re‐conceptualization of the nature of soil organic matter, are profoundly changing traditional views of SOC persistence (Lehmann & Kleber, 2015). These advances propose that the persistence of soil organic matter can emerge from the interaction between physicochemical and biological processes that simply reduce the probability of SOM decomposition and/or increase the probability of SOM accumulation (Schmidt et al., 2011). Therefore, SOC and SOM persistence is considered to be an emergent ecosystem property rather than the result of intrinsic chemical properties of SOM.

Lehmann and Kleber (2015) recently proposed the Soil Continuum Model (SCM) whereby SOM consists of an array of compounds covering the whole spectrum of large, undecomposed plant fragments, too small‐sized, easily decomposable monomers. Thermodynamically, the distribution of these compounds must follow a downhill energetic trajectory with more complex compounds progressively broken down into simpler and smaller compounds. As compounds become simpler and smaller, their reactivity toward minerals, and thus the likelihood of being protected within aggregates, increases. Also, the spatially heterogeneous distribution of SOM and microbes can physically disconnect SOM and decomposers (Schmidt et al., 2011). As argued by Lehmann and Kleber (2015), SOM and SOC are functions of the state of a dynamic system where decomposition and the formation of aggregates, and adsorption of simple and small compounds onto mineral surfaces, are balanced. As such, SOC is in a state of dynamic equilibrium whereby continuous losses are counterbalanced by continuous gains (Janzen, 2006, 2015). Under such conditions, SOC can remain at an equilibrium level over many decades, if not centuries (Johnston, Poulton, & Coleman, 2009). Overall, if conditions remain relatively stable, the persistence of SOC at this equilibrium level does not require the formation of recalcitrant SOM sequestering SOC permanently (Lehmann & Kleber, 2015), but just a balance between SOC losses and gains.

Here, we used the SCM concept to develop a dynamic equilibrium theory of SOC in which the persistence of SOC is the equilibrium point resulting from continuous SOC mineralization and gains due to C input and the extent to which SOM protection (e.g. aggregate formation) affects turnover rates. We aimed to show that assumptions of the soil continuum model can be translated into quantitative models that, in contrast to traditional SOC pool models, invoke neither a chemical stabilization nor the variable recalcitrance of discrete SOM pools. We fitted the model to 67 publicly available SOC time series obtained from 14 field experiments investigating the effects of agricultural practices on SOC in arable soils spanning timescales of 10–115 years. We first test whether the model can accurately describe the temporal variation of SOC starting from the idea that the temporal variation of SOC loss and gain rates are functions of SOC amounts (Six, Conant, Paul, & Paustian, 2002). We then show with this simple model that invokes neither recalcitrance nor distinct pools of SOC, that SOC persistence results from the ultimate equilibrium between SOC gains and losses, which may depend on environmental (e.g. temperature) and ecological (e.g. spatially structured microbial activities) factors and the relative roles of these factors. Our overarching goal was to examine SOC persistence based on the Soil Continuum Model to offer ecological insights for the next generation of experimental studies of SOC dynamics.

## 2 THEORETICAL CONCEPTS, ASSUMPTIONS, AND MODEL DERIVATION AND PARAMETERIZATION

In the framework of the SCM, the first step to developing an equilibrium theory of SOC dynamics is defining how rates of C loss and gain depend on soil state variables (i.e. the quantitative variables that describe the state of a soil system). There are many state variables that we could potentially start from, but the simplest is the amount of SOC. This choice is analogous to continuous population dynamic models where population growth rate is a function of population size (Chase, 2000). In the first instance, we assume that the instantaneous rate at which SOC is either lost or gained depends on the current state (i.e. quantity) of SOC. The use of SOC amounts as a state variable in C cycling models is justified by correlative evidence. For example, the rate of C accumulation (here “gain”) has been shown to be highly and negatively correlated with SOC in agricultural fields following land abandonment (Knops & Tilman, 2000), while estimated rates of organic C loss across English and Welsh soils over the period 1978–2003 were found to be positively correlated with SOC, although the results might be biased by the presence of highly organic soils in the data set (Bellamy, Loveland, Bradley, Lark, & Kirk, 2005; but see Schulze & Freibauer, 2005).

In general terms, the idea that the rate of change in SOC is a function *F* of SOC (C_soil_) can be expressed as

$$\frac{dC}{\mathrm{dt}}=F\left(C_{\mathrm{soil}}\right)$$

but we want to distinguish between rate of loss

$$\frac{dC_{\mathrm{loss}}}{\mathrm{dt}}=L\left(C_{\mathrm{soil}}\right)$$

and rate of gain

$$\frac{dC_{\mathrm{gain}}}{\mathrm{dt}}=\;G\left(C_{\mathrm{soil}}\right),$$

with *L* and *G* being the functions that respectively express how rates of loss and gain depend on the state variable C_soil_. We can therefore define the total rate of change as the difference between gain *G* and loss *L*, that is,

(1) $$\frac{dC}{\mathrm{dt}}=G\left(C_{\mathrm{soil}}\right)-\;L\left(C_{\mathrm{soil}}\right)$$

and consider that at equilibrium there is no net change in SOC because rate of gain *G* equals rate of loss *L*, so that

(2) $$G\left(C_{\mathrm{soil}}\right)\equiv L\left(C_{\mathrm{soil}}\right).$$

**Figure 1:**
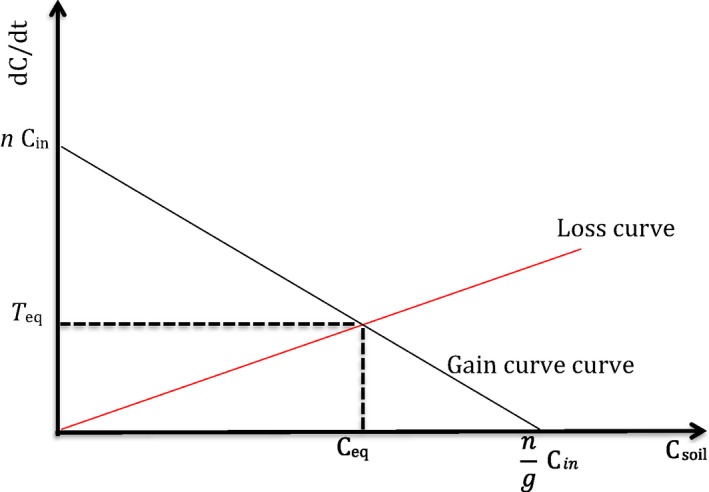
The simplest formulation of our model assumes linear curves for rate (*y*‐axis) of C loss (positive slope, solid red line) and gain (negative slope, solid black line), which are a function of the state variable C (*x*‐axis). The intersection point of the two curves gives the equilibrium (C_eq_) on the *x*‐axis and the turnover (*T*_eq_) on the *y‐axis*. At this point, soil organic carbon is in balance between two processes: aggregate formation, mineral adsorption, and formation of soil biota biomass; aggregate destruction, desorption, and respiration

If the functions *G* and *L* in Equations [Link] and [Link] were known and relatively simple (e.g. linear), it would be possible to solve for the equilibrium value of C (C_eq_). With the above assumptions in mind, we propose that the simplest equations regulating organic C losses and gains in soil could be

(3) $$\frac{dC_{\mathrm{loss}}}{\mathrm{dt}}=k\;C_{\mathrm{soil}}$$

(4) $$\frac{dC_{\mathrm{gain}}}{\mathrm{dt}}=n\;C_{\mathrm{in}}-g\;C_{\mathrm{soil}}$$

where *k, n*, and *g* are intrinsic rates per unit of time (typically in year) and C_in_ is the amount of C entered into the soil per unit of time (i.e. input C). For example, in an experiment with organic manures, C_in_ would correspond to the amount of C added to soil through the amendment. Equation [Link] is consistent with basic (i.e. 1st order kinetics) models of decay rate. Equation (1) expresses that when there is very little or no organic C in soil (e.g. at the start of primary succession), the rate of C gain is maximum and is proportional to the C entering the soil (*n* C_in_). However, as the soil starts to accumulate C, the maximum rate of gain will be reduced by a quantity that is proportional to SOC (*g* C_soil_). If soil accumulated all C entering at the start of the dynamics, final SOC would equal $\frac{nC_{\mathrm{in}}}{g}$ but this value will never be reached because C loss starts to become significant when SOC builds up. The dynamics resulting from coupling Equations [Link] and (1) are almost equivalent and mathematically very similar to a traditional one‐pool model (Falloon & Smith, 2009) with overall decay rate equal to the sum of our *g* and *k* rates. However, as we show below, there is major conceptual difference between the traditional and our approach, namely our distinction between rates of losses and gains. This distinction is critical to the development of SOC models derived from the soil continuum model because it is based on the main assumption that SOM is a continuum rather than consisting of discrete pools. Further, this distinction is critical because of our assumption that SOC models are independent of intrinsic chemical recalcitrance properties of SOM. The conceptual difference between rates of SOC loss and gain becomes clear when we represent the two components (i.e. SOC loss and gain rate curves) of the simplest possible model in a graph with SOC (C_soil_) on the *x*‐axis and the rate of change in SOC on the *y*‐axis (Figure 1): the loss curve (Equation [Link], red line in Figure 1) has a positive slope while the gain curve (Equation (1), black line in Figure 1) has a negative slope with an intercept equal to the maximum gain rate (which would be observable only when SOC is virtually zero). The two curves intersect at the equilibrium point, with “C_eq_” on the *x*‐axis and “Turnover rate *T*_eq_” on the *y*‐axis. At equilibrium, losses and gains continue to occur but because they are equal, SOC is at a stable value while C is continually turned over at some rate *T*_eq_. Theoretically, a mechanistic understanding of the controls on C dynamics is equivalent to experimentally identifying the abiotic and biotic factors that determine the slopes of the gain and loss curves. These curves do not need to be linear as nonlinear curves may also imply multiple equilibria. Here, we limit ourselves to show the simplest possible formulation of our model and focus on a theoretical discussion of our results and assumptions, and how the latter allow deriving models that can describe SOC dynamics well.

When the gain and loss curves are linear, simple algebra, and geometric considerations (Supporting Information Appendix S1, part *a*) show that

(5) $$C_{\mathrm{eq}}=\frac{n}{g+k}C_{\mathrm{in}}$$

(6) $$T_{\mathrm{eq}}=\frac{k\;n}{g+k}C_{\mathrm{in}}$$

where C_eq_ is C at equilibrium and *T*_eq_ is C turnover at equilibrium. The full solution of the dynamic model (for derivation see Supporting Information Appendix S1, part *b*) is

(7) $$C_{\mathrm{soil}}\left(t\right)=\frac{n}{g+k}\;C_{\mathrm{in}}\;-\;\frac{n\;C_{\mathrm{in}}-C_{0}\left(g+k\right)}{g+k}e^{-\left(g+k\right)\left(t-t_{0}\right)}.$$

**Figure 2:**
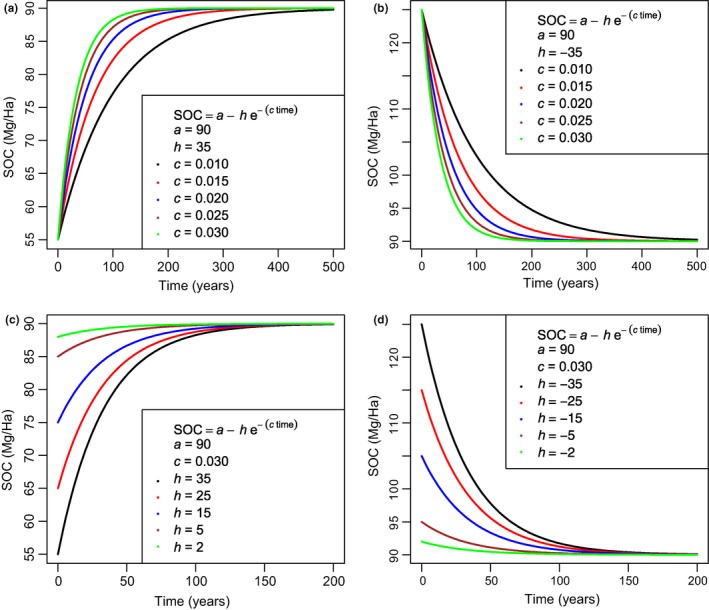
Effects of parameter *h* and *c* on soil organic carbon (SOC) dynamics. In (a,b) parameter *c* is varied (different colors) for positive and negative *h,* respectively. These show how *c* regulates the velocity at which SOC reaches its equilibrium value “*a*”. They also show that positive *h* is observed when initial SOC is lower than equilibrium (panel *a*) while negative *h* corresponds to initial SOC higher than equilibrium (panel *b*). In (c,d) the effect of varying *h* is shown for positive and negative values of *h,* respectively. Panels (c,d) illustrate how *h* mostly reflects initial conditions, especially the difference between initial SOC and equilibrium SOC

The solution of Equation (4) provides a nonlinear model (Supporting Information Appendix S1, part *c*) that can be statistically tested on SOC time series as

(8) $$\text{SOC}=a\;-h\;e^{-c\;t},$$

where *t* is time, equilibrium $a=C_{\mathrm{eq}}=\frac{n}{g+k}C_{\mathrm{in}}$, $h=\frac{n\;C_{\mathrm{in}}-C_{0}\left(g+k\right)}{g+k}$, and *c* equals the sum of intrinsic rates of loss *k* and gain *g* (compare Equations (4) and (5) to relate the compound parameters *h* and *c* to the fundamental parameters of the loss and gain curves). In practice, the *c* parameter regulates SOC turnover at equilibrium and the velocity at which SOC achieves the equilibrium value (Figure 2a,b). In this simple formulation of the model, if at initial time *t*_0_, SOC equals C_0_ and is smaller than equilibrium SOC (i.e. C_0_ *< *C_eq_) as it may happen during a succession or restoration process, SOC will increase monotonically until equilibrium is reached, which corresponds to scenarios where the parameter *h* is positive (Figure 2a,c). Vice versa, if the initial equilibrium point decreases because conditions are changed by some disturbance factor (e.g. change from no to conventional tillage) SOC will for a period of time be higher than the new equilibrium and will decrease exponentially until it reaches its new equilibrium value: in this case, *h* is negative (Figure 2b,d). These two examples show that the parameter *h* reflects initial conditions (specifically, C_0_ relative to C_eq_). The effect of changing model parameters can be explored using the R script provided in the Supporting Information Data S2.

The nonlinear model of Equation (5) can be fitted to experimental time series of SOC. In these time series, stocks of SOC are commonly expressed either as t/ha to a certain soil depth (i.e. Mg/ha in SI), g/kg, or mg/g (Supporting Information Data S1). Information on soil bulk density (g/cm^3^) and soil depth (cm) can be used to convert SOC stocks from unit of area (ha^−1^) to unit of soil weight (kg^−1^ or g^−1^). In the literature, rates of SOC loss and gain are typically expressed in year^−1^. For example, rate of loss *L*(C_soil_) or turnover rate at equilibrium *T*_eq_ can be expressed as t ha^−1^ year^−1^ or g kg^−1^ year^−1^. Accordingly, the rate parameters *k*,* g*, and *n* of our model can be expressed in unit of time^−1^ as year^−1^. Once units have been defined, the nonlinear model of Equation (5) can be fitted to time series to estimate the value of the key, individual parameters *k*,* g*, and *n*, which are embodied in the parameters *h* and *c* of the nonlinear model in Equation (5). First, once *h* and *c* are estimated by fitting this version of the model (Equation (5) to the time series, parameter *n* can be estimated because C_0_ and C_in_ are usually known. C_0_ is the initial SOC and C_in_ (C in input) is usually known in experimental plots. For example, in farmyard manure treatments the amount of organic C added with manure may be around 3 t ha^−1^ year^−1^. Then, the parameters *g* and *k* can be estimated as follows: when initial SOC is well below equilibrium SOC (C_0_ ≪ C_eq_; e.g. Figure 2a), the initial dynamic is dominated by C gain, which allows an estimate of *g* using Equation (1). When initial SOC is well above the equilibrium (C_0_ ≫ C_eq_; e.g. Figure 2b), the dynamic is initially dominated by C loss, and *k* can be estimated using Equation [Link] on the first part of the time series. The estimate of one of these two rates (either *k* or *g*) allows the estimate of the other rate via the parameter *c,* which equals the sum of *k* and *g*.

## 3 TESTING THE MODEL

We used a publicly available database created for the Swedish Foundation for Strategic Environmental Research (http://www.eviem.se/en/projects/Soil-organic-carbon-stocks/). This global database is linked to a systematic GIS map of 735 scientific publications reporting data on the effects of agricultural practices on SOC of arable soils (Haddaway et al., 2015). Each study is georeferenced on a global map and most studies are accessible via Google Scholar. Metadata associated with the database allowed us to search for adequate time series. Specifically, we filtered the records for studies having times series with at least six data points covering 10 years, and we then searched each paper for figures or tables that could be used to recover the time series data points. Eventually, we used 11 papers from the database for a total of 60 time series data sets (Buysse, Roisin, & Aubinet, 2013; Campbell, Zentner, Selles, Liang, & Blomert, 2001; Clapp, Allmaras, Layese, Linden, & Dowdy, 2000; Fließbach, Oberholzer, Gunst, & Mäder, 2007; Gan et al., 2012; Heenan, Chan, & Knight, 2004; Hendrix, Franzluebbers, & McCracken, 1998; Hernanz, Sánchez‐Girón, & Navarrete, 2009; Kätterer, Börjesson, & Kirchmann, 2014; Seremesic, Milosev, Djalovic, Zeremski, & Ninkov, 2011; Zanatta, Bayer, Dieckow, Vieira, & Mielniczuk, 2007). In addition, we used seven long‐term time series of SOC from field experiments conducted at Rothamsted, England (Johnston et al., 2009). All 67 time series we analyzed were obtained from experiments (see Supporting Information Data S1) investigating how SOC changes over time in response to various combinations of fertilizer (e.g. farmyard manure vs. mineral N and P), tillage (e.g. conventional vs no tillage), management (e.g. organic vs. conventional farming), and rotation regimes. We tested the nonlinear model of Equation (5) on these 67 real‐time series using the function nls in the R package nlme (see Supporting information for R script and Supporting Information Data S3; DataSet.txt; see also further information provided in Supporting Information Appendix S1).

**Figure 3:**
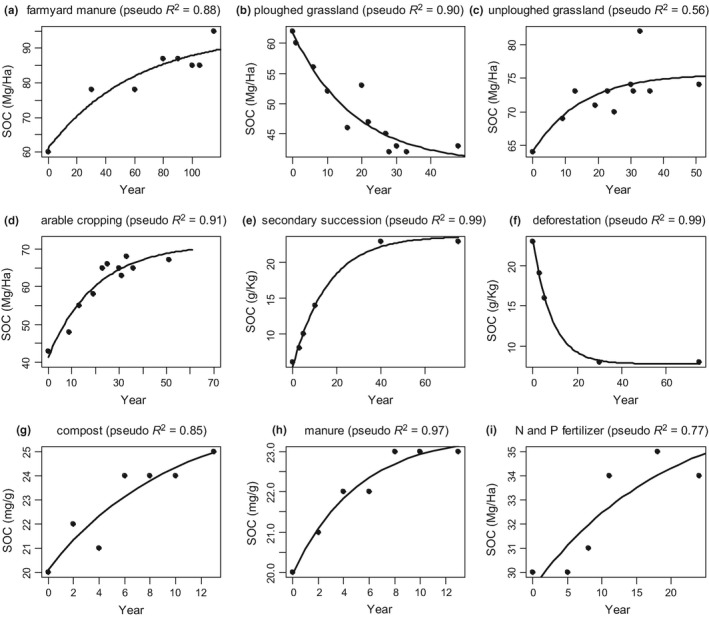
Nine examples of model fit to real‐time series (full set is given in the Supporting Information Data S3). Time series (a–d) are from Johnston et al. (2009) and come from: (a) farmyard manure applied annually since 1885; (b) long‐term grassland subjected to ploughing; (c) unploughed long‐term grassland; (d) arable cropping under newly sown grass. Hendrix et al. (1998) offered an interesting comparison of: (e) a natural succession process after the conversion of degraded soils from row cropping to sod or kudzu (*Pueraria lobata*); (f) and the plowing of native forest soil followed by long‐term, intensive row cropping. Kätterer et al. (2014) reported the effects of various types of fertilizers and here we show their series for: (g) compost obtained from domestic waste; (h) and manure from a from a cowshed with straw bedding. Finally, in (i) a SOC time series from a fallow‐wheat rotation with N and P fertilizers as reported in Gan et al. (2012). The coefficient of determination was calculated using a pseudo‐*R* ^2^ (also known as generalized *R* ^2^; Nagelkerke, 1991), which is based on a null model to estimate model log‐likelihood ratio

The formulation of the model as shown in Equation (5) could be fitted to 39 out of 67 time series (Supporting Information Data S1). These 39 time series showed a simple monotonic increase or decrease in SOC toward a theoretical equilibrium SOC, implying relatively constant abiotic and biotic conditions (i.e. SOC equilibrium point is not changing over time) and initial SOC higher or lower than equilibrium SOC (i.e. SOC changes over time to reach the equilibrium point). More complex multi‐pool models, but also one‐ or two‐pool C models, are likely to fit the same data equally well or better, but our aim here was not to provide a better alternative to current SOC models. Sufficiently complex mathematical models will always describe SOC temporal dynamics to a high degree of accuracy and precision, and a large number of models can be fitted to any SOC time series until the best fitting model is found, regardless of model assumptions. Yet, we aimed to examine whether a simplistic formulation of our model fit a range of SOC time series because we wanted to offer a first quantification of the soil continuum model and propose new directions for SOC modeling. Indeed, and as expected, the specific formulation of our model as in Equation (5) could not be fitted successfully to time series obtained under experimental regimes whereby treatments caused important and confounding changes in conditions over time (e.g. rotation; Supporting Information Data S1). In a few cases, some treatment combinations had very little effect on SOC, which might have fluctuated around the equilibrium point, as for instance observed in some of the time series reported by Heenan et al. (2004) (see details in Supporting Information Data S1). When the model could be fitted, we found that model fit was very good, and in most cases excellent (see examples Figure 3. Full results are given in Supporting Information Data S1), especially considering the noise potentially observable in long‐time series data of SOC in arable soil. Nevertheless, as hypothesized by Johnston et al. (2009), most of the studies we investigated (Figure 3, Supporting Information Data S1) provided clear evidence that SOC eventually reaches an equilibrium point or fluctuates around an average value that might be interpreted as the equilibrium point.

The parameter estimates obtained from the time series used in this study (Supporting Information Data S1) show that rates of loss and gain vary between 0.001 and 0.1 year^−1^, although more extreme values are possible.

## 4 DETERMINANTS OF LOSS AND GAIN RATES AND FUTURE DIRECTIONS

**Figure 4:**
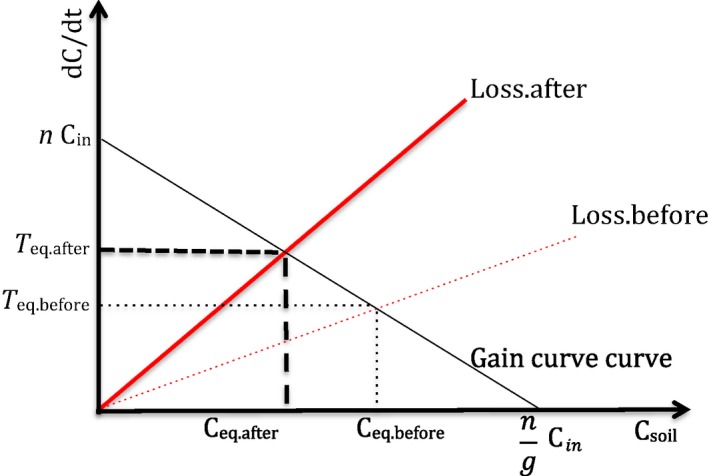
A scenario in which soil organic carbon (SOC) responds to a perturbation just by increased loss rates. This means that the slope of the loss rate curve (red line) increases (compare dotted and solid red lines, which correspond to loss rate before and after perturbation, respectively). This change moves the equilibrium point leftwards, with C_eq.after_ < C_eq.before_: The increase in loss rate is not balanced by an increase in gain rate and overall the systems are also accelerating SOC turnover (*T*_eq.after_ > *T*_eq.before_)

Temporal variation in SOC loss and gain drives the dynamics of SOC, but many factors can determine this variation. In the basic version of the model, SOC dynamics depend on the mathematical shape of the functions that describe the rates of loss and gain. In the framework proposed here, a mechanistic understanding of the SOC dynamics coincides with an experimental investigation of the factors that determine the shape of the gain and loss curves. This shape can be complex, but here we just illustrate the equilibrium approach using the simplest possible model formulation. More complex models, either traditional ones or the equivalent ones we could derive from our approach, can certainly fit the data presented better than the simplistic version illustrated here. However, the simple formulation we present here and our conceptual approach may offer new perspectives on the processes that determine SOC variation in soil. For example, the factors that drive SOC loss and gain can be introduced as either categorical or continuous variables that affect the shape of the SOC loss and gain curve over time, space, and/or treatments. Any model, including ours and traditional models using conceptual pools, cannot provide mechanistic insights in their own right. But if models are coupled with appropriate experiments then they can help to tease apart the factors that mechanistically control SOC dynamics. For example, in the case of our model, let us consider the simplest possible formulation (Figure 1) and assume that the loss and gain curve were both linear functions of SOC (Equation [Link] and (1)). Under this assumption, the abiotic and biotic factors that affect loss and gain can simply be introduced as variables that change the slope of the SOC loss and gain lines (Figure 4) and experiments can be designed to quantify the effect of these factors.

The major conceptual difference between our approach and the more traditional ones, such as one‐pool or two‐pools models, is that in our case the persistence of SOC emerges from the difference between loss and gain and the dynamic equilibrium that maintains this difference, regardless of whether and which factors affect either loss or gain, or both. For example, tillage is known to reduce the abundance of earthworms, arbuscular mycorrhizal, and decomposer fungi, root‐feeding fauna and their predators, with direct and indirect effects on rates of C loss and gain (Postma‐Blaauw, de Goede, Bloem, Faber, & Brussaard, 2010; Tsiafouli et al., 2015; de Vries et al., 2013). Regardless of the specific mechanisms via which tillage affects soil physical and chemical properties and biota, we can use our model to describe the impact of tillage as an effect on the slope of either the loss or the gain curve (Figure 4). In the example of tillage, we could either assume that the mechanical perturbation caused by tillage increases rate of SOC loss over time or decreases the rate of SOC gain. Actual experiments can resolve which mechanism is dominant in specific contexts.

Let us hypothesize that tillage causes detrimental changes in soil biotic and abiotic conditions, and that these changes increase the slope of the loss curve as shown in the *Loss.after curve* versus *the Loss.before curve* (Figure 4). This change moves the equilibrium point to the left (lower SOC: C_eq.after_ < C_eq.before_) because an increase in loss rate with no increase in gain rate must accelerate turnover (*T*_eq.after_ > *T*_eq.before_) via losses. The hypothesis that tillage accelerates SOC turnover or lowers the equilibrium SOC could be tested by fitting a “blocked” version (Supporting Information Appendix S1) of the nonlinear model in Equation (5)) to time series obtained during investigation of the impact of tillage on SOC persistence. Blocks are different experimental groups. The blocked model works as follows: the factor “tillage”, which could consist of various levels (e.g. control, moderate, intense), could be explicitly introduced in the model as a categorical variable that controls the average value of the model parameters *a* (equilibrium), *c* (loss and gain rates), and *h* (input and initial condition) in Equation (5). Other experimental factors such as organic matter application or land use conversion could be investigated likewise (see for example the comparison offered in Figure 3e,f, which reports two‐time series from a study of land use conversion and the model fit). Thus, experimental factors that drive the values of model parameters are relatively straightforward to introduce in the simple formulation of Equation (5).

**Figure 5:**
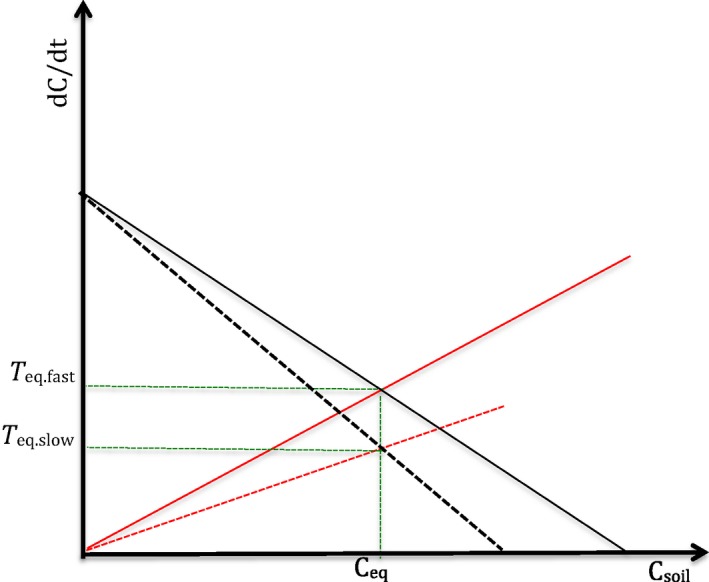
High turnover is not incompatible with high C at equilibrium if both loss and gain rates curve change in a way that increased losses are compensated by increased gains. Curves represented by solid black (gain) and red (loss) lines represent a new, faster state relative to those shown as dotted black (gain) and red (loss) lines. Still, because both gain and loss rates are increasing, the system still stays at the same C at equilibrium while having a higher Turnover. This scenario is consistent with recent empirical observations that show how systems with higher turnover might still have high SOC (Bender et al., 2016; de Vries et al., 2013)

The example of tillage conceptually demonstrates the ability of the equilibrium idea to accommodate scenarios in which the distinct processes of SOC loss and gain vary as a function of the biotic and abiotic factors that determine SOC loss and gains. Scenarios with high turnover rate and high SOC are also possible (Figure 5, see also Supporting Information Appendix S1 Part C). Additionally, the slope of the loss and gain curves can also be made function of continuous abiotic and biotic variables (e.g. microbial biomass), which also vary over time. Also, the loss and gain curves could be nonlinear or could have time‐dependent slopes. Equilibrium SOC can then be made to fluctuate over time, depending on the fluctuations of the variables driving the rate of SOC loss and gain. Possible examples of these scenarios and how to change the model equations are offered in the Supporting Information Appendix S1 (part *c*) but the general point is that the mathematical complexity of the model can be increased to accommodate scenarios in which: (a) equilibrium SOC varies over time because of temporal variation in the rates of either SOC gain or losses; (b) there are multiple determinants of both SOC gain and losses. The simplest formulation of our model could fit 39 out of the 67 time series used in the present analysis and in some cases the fit was excellent (see for example the nine cases illustrated in Figure 3). This suggests that our process‐based idea of equilibrium is a promising starting point for the formulation of future SOC models, besides the incorporation of the specific mechanisms that determine the rates of SOC gain and loss. The simple version of the model does not account for variation in abiotic and biotic conditions over time, which determines unaccounted variation in rates of SOC gain and loss and may explain the failure of the model in fitting some of the time series data considered here. Another important reason is that some of the observed time series may actually display erratic variation around the equilibrium SOC simply because the experimental treatments did not exert the effects which were meant to be exerted. A further investigation of this variation should clarify the mechanisms underpinning this seemingly erratic variation (e.g. seasonal fluctuation in microbial community structure and activity, and OM input). Once these mechanisms are identified they can be incorporated into the functional shape of the gain and loss functions in order to formulate models that can better reflect the processes determining SOC variation over time and thus accommodate specific patterns of temporal fluctuations in SOC. More generally, the conceptual framework we have introduced here can in the future accommodate the actual, multivariate complexity that regulates SOC dynamics (Falloon & Smith, 2009; Sihi, Gerber, Inglett, & Inglett, 2016; Sihi, Inglett, Gerber, & Inglett, 2018), including, but not limited to, plant–soil interactions in the context of global change over multiple scales (Bardgett, Manning, Morrien, & Vries, 2013; van der Putten et al., 2013) and SOC spatial heterogeneity as linked to microbial dynamics (Kaiser, Franklin, Richter, & Dieckmann, 2015; Lehmann et al., 2008; Lehmann & Kleber, 2015).

## 5 CONCLUSION

We show that, in line with recent conceptual advances in soil organic matter theory, variation in the rate of SOC loss and gain, and their balance over time, can be modeled effectively as a direct function of SOC itself, without invoking processes of chemical stabilization of SOM and the property of SOM recalcitrance, which underpin current dynamic models of SOC. Our model also allows incorporating the influence of abiotic and biotic factors to test and model ecological mechanisms that determine SOC dynamics. In addition, our model offers a quantitative framework for future experiments aimed at quantifying the relative roles of the ecological drivers of SOC dynamics across spatial and temporal scales.

## AUTHORS’ CONTRIBUTIONS

All authors developed the concept of the paper; TC formulated theoretical and mathematical models, compiled the data, and performed statistical analyses. All authors contributed substantially to the writing of the manuscript.

## DATA ACCESSIBILITY

Data available from the Dryad Digital Repository: https://doi.org/10.5061/dryad.ch10ss4
