# Supplementary material for: Soil organic carbon dynamics matching ecological equilibrium theory
Source: Ecol Evol. 2018 Oct 18;8(22):11169–78. doi: 10.1002/ece3.4586 (PMC6262907; doi:10.1002/ece3.4586)
Supplement: Supplementary file 1 [file ECE3-8-11169-s001.docx]

**Supplementary Information**

*a) Equilibrium and turnover*

We start from the loss and gain equations:

$\frac{{dC}_{loss}}{dt}=k C_{soil}$

$\frac{{dC}_{gain}}{dt}=n C_{in}-{g C}_{soil}$

and express the rate of variation of *C* in soil (SOC) as the difference between the rates at which *C* is gained and lost, that is

$$\frac{{dC}_{soil}}{dt}=nC_{in}-{gC}_{soil}- kC_{soil}$$

At equilibrium the net rate of change is zero and so we can solve for organic C in soil as follows:

$\frac{{dC}_{soil}}{dt}=0\to0=nC_{in}-{gC}_{soil}- kC_{soil}$

leading to

$$Ceq=\frac{n}{g+k}Cin$$

Turnover is the value of either loss or gain rate, which are never null but just equal at equilibrium. To find this value, we need to solve for either the rate of loss or that of gain when C_soil_ equals *C*_soil.eq._. For example, if we use the simpler loss curve we obtain

$$\frac{{dC}_{loss}}{dt}=k \left( \frac{n}{g+k} \right)C_{in}=Teq$$

*b) Full Solution*

We can fully solve the ordinary differential equation for SOC

$$\frac{{dC}_{soil}}{dt}=nC_{in}-{gC}_{soil}- kC_{soil}$$

by separation of variables (Robinson 2004), which leads to:

$$\int_{C0}^{C} \frac{1}{nC_{in}-(g+ k{)C}_{soil}} {dC}_{soil}=\int_{t0}^{t} dt$$

The right hand side is obvious, the indefinite solution being just *t* plus a constant.

To solve the left hand side, we use the following well known indefinite integral:

$$\int\frac{1}{a-b x} dx=-\frac{\log(a-b x)}{b}$$

And we take *nC_in_ = a*, *(g+k) = b,* and *Csoil* = *x* being the variable of integration. That leads to

$$\left| \left( -\frac{log({nC}_{in}-\left( g+ k \right)C)}{(g+k)} \right)-\left( -\frac{log({nC}_{in}-\left( g+k \right)C0)}{(g+k)} \right) \right|=\left| t-t0 \right|$$

which by rearranging terms and exponentiation becomes:

$$\frac{{nC}_{in}-\left( g+k \right)C0}{{nC}_{in}-\left( g+k \right)C}=e^{(t-t0)(g+k)}$$

in which *C* is SOC at time t, or *C_soil_(t).* By rearranging the terms to resolve for *C* we then obtain the full solution:

$$Csoil\left( t \right)=\frac{n}{g+k}Cin-\frac{n Cin-C0(g+k)}{g+k}e^{-(g+k)(t-t0)}$$

*c) Nonlinear statistical models*

The full solution expresses SOC as a function of time and is rewritable in a general way as

$$SOC=a -he^{-c t}$$

Where *a =* $\frac{n}{g+k}Cin$ *,* h = $\frac{n Cin-C0(g+k)}{g+k}$*and c = (g+k),* while *C*, *t* and *e* are obviously carbon, time and the Napier number (exponentiation).This is a saturation function levelling off at *a*, the equilibrium SOC level. The rate at which the function levels off is fundamentally governed by *c,* which is the sum of g and k, the intrinsic rates of C gain and loss respectively. Over time, the exponential term tends to zero, and C settles at *a*.

Any time series can be fitted with this model, which is particularly intuitive in a situation where soil is initially well below its equilibrium but then builds up over time to reach equilibrium. The nonlinear model is easy to fit to times series of SOC, where *C* (SOC) is the dependent variable *x* and time is the independent variable *y*. For example, in R using the function nls in package nlme one can fit the model as follows (Ritz & Streibig 2008 see also Supplementary File R_Script)

*library(nlme)*

*ModelFit1<- nls(Cdata ~ a-h*exp(-c*time),data = datamodel, start = list(a = 25, h = 0.08, c = 2))*

In this line of code, we assume that data are stored in the data framework “datamodel”, and the list of initial parameter values is based on a preliminary estimate, which is usually based on a quick inspection of the data (basically, in this case by plotting *C* against time).

The interesting aspect is that one can easily expand the model to include factors and variables that might modify model parameters. For example, g and k might depend on the fungal to bacterial ratio or one may introduce the effect of particular treatments on the parameters (e.g., management types such as extensive vs intensive).

Assume two time series from the same area: in time series “In”, soil was under intensive management, while in time series “Ex” soil was under extensive management. Data are thus grouped by the factor “management” (*Man*), which consists of two levels (intensive vs extensive). We can then test the hypothesis that management affects gain and loss rates by fitting a nonlinear model as follows:

*ModelFit_Groups<- nls(Cdata ~ a[Man]-(h[Man])*exp(-c[Man]*time),data = datamodel, start = list(a = c(25,35) h = c(0.08,0.09), b = 2))*

Where *a[Man]* and *h[Man]* means that model parameters may differ between the two levels of the factor *Man*, that is between *In* and *Ex*. In the initial parameter values, for example, we guess that *In* might be around 25 t C ha^-1^ while *Ex* around 35 t C ha^-1^. Random effects due to location or plot can be introduced in this statistical modelling framework by using the facilities provided in functions such as nlme (Pinheiro & Bates 2000).

C) Examples of continuous determinants of gain and loss rates

The relative abundance of fungi and bacteria has for a long time been considered a key soil food web property that responds to soil management (e.g., intensive vs. extensive) and correlates with processes of C and N cycling (Hendrix *et al.* 1986; de Vries *et al.* 2006; Kallenbach *et al.* 2016). Shifts in the relative abundance of fungi and bacteria could be introduced in the model to regulate rates of loss and gain, the key point being that soil with a high fungal to bacterial ratio are usually characterised by high SOC (Hendrix *et al.* 1986; de Vries *et al.* 2006, 2013), even when these soils might have relatively high soil respiration rates (e.g., de Vries *et al.* 2013). It is also possible that higher SOC determines higher biomasses of fungi and bacteria and only experiment can resolve the direction of causality. For simplicity, let’s assume that the fungal to bacterial ratio regulates rates of SOC losses. More specifically, some literature suggests that a lower fungal to bacterial ratio is correlated to an increase of SOC loss rates (Malik *et al.* 2016), which could in the first instance be modelled linearly as follows

$$\frac{{dC}_{loss}}{dt}=\frac{b}{f}k' C_{soil}$$

*b/f* being the bacterial to fungal ratio. This equation suggests that the higher the fungal to bacterial ratio the lower the C loss. The fungal to bacterial ratio could, however, also increase SOC gains, as shown below. In any case, an important point to note in the context of the relationship between fungal to bacterial ratio, SOC, and soil respiration, is that in our model high equilibrium SOC can be maintained at a high rate of SOC turnover as long as high rates of SOC losses are compensated by high rates of SOC gains (see figure 5 in main text). Such a system can maintain high SOC at high turnover rates: a fast turnover of SOC does not necessarily imply net SOC losses via increased respiration or leaching. A possible mechanism through which losses can be compensated by gains due to increased fungi is the formation of soil structure. In fact, increased fungal biomass usually correlates with improved soil structure (TIsdall & Oades 1982; Six *et al.* 2004; Rillig & Mummey 2006). Consequently, a simple way to introduce SOC gain via soil structure could be

$\frac{{dC}_{gain}}{dt}=n C_{in}-{\frac{g'}{d} C}_{soil}$

*d* being an index of soil structure such as mean weight diameter or the exponent of the power laws that describe the relationship between soil particles size and numbers (e.g., Caruso *et al.* 2011).

More generally, we can write

$\frac{{dC}_{loss}}{dt}=X\left( \frac{f}{b} \right) C_{soil}$

$$\frac{{dC}_{gain}}{dt}=n C_{in}-{Y(d) C}_{soil}$$

were *X* and *Y* are respectively functions of *f/b* (fungal to bacterial ratio) and *d* (soil structure). There are numerous potential shapes for the generic functions *X* and *Y*, which will need to be investigated in the future and will depend on the mechanistic resolution that future experimental studies may allow to achieve. Still, the mathematical formalism that introduces these general functions helps with conceptualising existing information to develop a new modelling framework. In the future, this framework will have to accommodate the actual, multivariate complexity known to regulate SOC dynamics. The general point is that the loss and gain equations can have functional shapes of any complexity to account for complex patterns of temporal variation in SOC.

References

Caruso, T., Barto, E.K., Siddiky, M.R.K., Smigelski, J. & Rillig, M.C. (2011). Are power laws that estimate fractal dimension a good descriptor of soil structure and its link to soil biological properties? *Soil Biol. Biochem.*, 43, 359–366.

Falloon, P. & Smith, P. (2009). Modelling soil carbon dynamics. In: *Soil Carbon Dynamics: An Integrated Methodology*. Kutsch W.L., Bahn M., Heinemeyer A. Cambridge University Press, pp. 221–244.

Hendrix, P.F., Parmelee, R.W., Crossley, D., Coleman, D.C., Odum, E.P. & Groffman, P.M. (1986). Detritus food webs in conventional and no-tillage agroecosystems. *Bioscience*, 36, 374–380.

Kaiser, C., Franklin, O., Richter, A. & Dieckmann, U. (2015). Social dynamics within decomposer communities lead to nitrogen retention and organic matter build-up in soils. *Nat Commun*, 6.

Kallenbach, C.M., Frey, S.D. & Grandy, A.S. (2016). Direct evidence for microbial-derived soil organic matter formation and its ecophysiological controls. *Nat. Commun.*, 7, 13630.

Lehmann, J. (2008). Spatial complexity of soil organic matter forms at nanometre scales. *Nat. Geosci*, 1, 238–242.

Lehmann, J. & Kleber, M. (2015). The contentious nature of soil organic matter. *Nature*, 528, 60–68.

Malik, A.A., Chowdhury, S., Schlager, V., Oliver, A., Puissant, J., Vazquez, P.G.M., *et al.* (2016). Soil Fungal:Bacterial Ratios Are Linked to Altered Carbon Cycling. *Front. Microbiol.*, 7, 1247.

Pinheiro, J.C. & Bates, D.M. (Eds.). (2000). Nonlinear Mixed-effects Models: Basic Concepts and Motivating Examples. In: *Mixed-Effects Models in S and S-PLUS*. Springer New York, New York, NY, pp. 273–304.

van der Putten, W.H., Bardgett, R.D., Bever, J.D., Bezemer, T.M., Casper, B.B., Fukami, T., *et al.* (2013). Plant–soil feedbacks: the past, the present and future challenges. *J. Ecol.*, 101, 265–276.

Rillig, M.C. & Mummey, D.L. (2006). Mycorrhizas and soil structure. *New Phytol.*, 171, 41–53.

Ritz, K. & Streibig, J.C. (2008). *Nonlinear Regression with R*. Springer-Verlag, New York.

Robinson, J.C.. (2004). *An Introduction to Ordinary Differential Equations*. Cambridge University Press.

Six, J., Bossuyt, H., Degryze, S. & Denef, K. (2004). A history of research on the link between (micro)aggregates, soil biota, and soil organic matter dynamics. *Adv. Soil Struct. Res.*, 79, 7–31.

TIsdall, J.M. & Oades, J.M. (1982). Organic matter and water-stable aggregates in soils. *J. Soil Sci.*, 33, 141–163.

de Vries, F.T., Hoffland, E., van Eekeren, N., Brussaard, L. & Bloem, J. (2006). Fungal/bacterial ratios in grasslands with contrasting nitrogen management. *Soil Biol. Biochem.*, 38, 2092–2103.

de Vries, F.T., Thébault, E., Liiri, M., Birkhofer, K., Tsiafouli, M.A., Bjørnlund, L., *et al.* (2013). Soil food web properties explain ecosystem services across European land use systems. *Proc. Natl. Acad. Sci.*, 110, 14296–14301.
